# Sex differences in treatments and outcomes of patients with cardiogenic shock: a systematic review and epidemiological meta-analysis

**DOI:** 10.1186/s13054-024-04973-5

**Published:** 2024-06-06

**Authors:** Thomas Fisher, Nicole Hill, Antonis Kalakoutas, Assad Lahlou, Krishnaraj Rathod, Alastair Proudfoot, Alex Warren

**Affiliations:** 1 North Bristol NHS Trust, Southmead Rd, Bristol, BS10 5NB UK; 2 Royal United Hospitals Bath NHS Foundation Trust, Combe Park, Bath, Avon, BA1 3NG UK; 3 Barts Health NHS Trust, W Smithfield, London, EC1A 7BE UK; 4 Barts Health Library Services, W Smithfield, London, EC1A 7BE UK; 5 William Harvey Research Institute, Barts and The London Faculty of Medicine and Dentistry, Queen Mary University of London, W Smithfield, London, EC1A 7BE UK; 6 Critical Care and Perioperative Medicine Group, School of Medicine and Dentistry, Queen Mary University London, W Smithfield, London, EC1A 7BE UK; 7 Anaesthesia, Critical Care and Pain Medicine, University of Edinburgh, 51 Little France Crescent, Edinburgh, EH16 4SA UK

**Keywords:** Cardiogenic shock, Myocardial infarction, Mechanical circulatory support, Sex differences, Epidemiology

## Abstract

**Background:**

Women are at higher risk of mortality from many acute cardiovascular conditions, but studies have demonstrated differing findings regarding the mortality of cardiogenic shock in women and men. To examine differences in 30-day mortality and mechanical circulatory support use by sex in patients with cardiogenic shock.

**Main body:**

Cochrane Central, PubMed, MEDLINE and EMBASE were searched in April 2024. Studies were included if they were randomised controlled trials or observational studies, included adult patients with cardiogenic shock, and reported at least one of the following outcomes by sex: raw mortality, adjusted mortality (odds ratio) or use of mechanical circulatory support. Out of 4448 studies identified, 81 met inclusion criteria, pooling a total of 656,754 women and 1,018,036 men. In the unadjusted analysis for female sex and combined in-hospital and 30-day mortality, women had higher odds of mortality (Odds Ratio (OR) 1.35, 95% confidence interval (CI) 1.26–1.44, *p* < 0.001). Pooled unadjusted mortality was 35.9% in men and 40.8% in women (*p* < 0.001). When only studies reporting adjusted ORs were included, combined in-hospital/30-day mortality remained higher in women (OR 1.10, 95% CI 1.06–1.15, *p* < 0.001). These effects remained consistent across subgroups of acute myocardial infarction- and heart failure- related cardiogenic shock. Overall, women were less likely to receive mechanical support than men (OR = 0.67, 95% CI 0.57–0.79, *p* < 0.001); specifically, they were less likely to be treated with intra-aortic balloon pump (OR = 0.79, 95% CI 0.71–0.89, *p* < 0.001) or extracorporeal membrane oxygenation (OR = 0.84, 95% 0.71–0.99, *p* = 0.045). No significant difference was seen with use of percutaneous ventricular assist devices (OR = 0.82, 95% CI 0.51–1.33, *p* = 0.42).

**Conclusion:**

Even when adjusted for confounders, mortality for cardiogenic shock in women is approximately 10% higher than men. This effect is seen in both acute myocardial infarction and heart failure cardiogenic shock. Women with cardiogenic shock are less likely to be treated with mechanical circulatory support than men. Clinicians should make immediate efforts to ensure the prompt diagnosis and aggressive treatment of cardiogenic shock in women.

**Supplementary Information:**

The online version contains supplementary material available at 10.1186/s13054-024-04973-5.

## Background

Cardiogenic shock is a complex syndrome of systemic hypoperfusion resulting from cardiac dysfunction. The observed incidence of cardiogenic shock in the United States has tripled between 2004 and 2018 [1]. Acute mortality ranges between 30–50%, despite improvements in recognition and management [2–4]. While most studies of patients with cardiogenic shock have focused on acute myocardial infarction (AMI), cardiogenic shock caused by other pathologies has become the predominant aetiology in cardiac critical care units [4, 5].

Compared to men, women appear to have higher mortality in other acute cardiovascular pathologies such as out-of-hospital cardiac arrest [6] and ST-elevation myocardial infarction (STEMI) [7], but may have a similar or even better prognosis in heart failure [8, 9]. Similarly, women with severe acute respiratory failure have been found to be more likely to die and to be ventilated with potentially injurious ventilator settings [10].

Several studies using adjusted analyses to examine the effect of sex on outcomes in cardiogenic shock have been undertaken, with some reporting higher mortality in women [11, 12], and others no difference [13–15]. Similarly, registry data show that women are less likely to receive mechanical circulatory support (MCS) than men, despite observational data suggesting they may derive greater benefit [16].

In this systematic review and meta-analysis, we aimed to assess the relationship between sex with mortality and receipt of mechanical circulatory support, adjusting for confounding factors where possible.

## Methods

### Search strategy

This epidemiological systematic review and meta-analysis was pre-registered on PROSPERO (CRD42022380480). Throughout, we followed the Meta-Analysis for Observational Studies in Epidemiology guidelines [17]. We searched Cochrane Central, PubMed, MEDLINE and EMBASE for studies reporting sex-specific mortality and treatment in patients with cardiogenic shock up to 19 April 2024. The full search strategy was developed in conjunction with a medical librarian at Barts Health NHS Trust (A.L.) and is listed in the Supplementary Appendix.

Studies were included if they met both of the following criteria: (1) Observational study or randomised controlled trial enrolling adult patients with cardiogenic shock. (2) Reported by sex at least one of: (a) unadjusted in-hospital or 30-day mortality or longer-term outcome; (b) adjusted mortality at least in-hospital or 30 days (i.e. by odds ratio or risk ratio between men and women); (c) use of MCS devices in men and women. Studies in languages other than English were included with translation as required. Case reports and non-human studies were excluded.

Mortality was extracted preferentially as 30-day mortality, then as in-hospital mortality if 30-day mortality was not reported. Studies which reported only shorter-term outcomes (e.g. ICU mortality) were not included. Studies which reported only longer-term outcomes were included only if in-hospital mortality/30-day mortality were not reported, as epidemiological studies have demonstrated that patients with cardiogenic shock who survive critical illness have relatively low rates of mortality in the first year following admission, comparable to similarly morbid patients [18, 19]. Studies reporting cohorts of patients receiving a particular treatment were included only if all, or a clear, separately reported cohort, had cardiogenic shock.

The search was carried out using Covidence software (Veritas Health Innovation, Melbourne, Australia) under licence from the University of Edinburgh. After importation of references, titles and abstracts were screened by three researchers (T.F., N.H. and A.K.). Arbitration of discrepancies and full-text review was undertaken by a separate researcher (A.W.). Multiple publications from the same dataset or study were identified manually. In order to prevent bias from multiple inclusion, only the most comprehensive study (i.e. with the lowest risk of bias, broadest acceptable inclusion criteria, longest search period and largest sample size) was included.

Bias was assessed for all studies with the Newcastle–Ottawa Score for observational cohort studies [20] by at least two of three independent researchers (T.F., N.H., A.K.) and arbitration by a fourth (A.W.). Randomised studies were assessed in the same way, given the objective was to assess the sex-related epidemiological data reported rather than bias of the randomised intervention. Definition for low risk of bias was Newcastle–Ottawa Score of ≤ 7.

### Statistical analysis

Three separate analyses were conducted for the outcomes of unadjusted mortality, adjusted mortality, and mechanical cardiac support use. Data were extracted for: study size, location, study type, population, number of men, number of women, total number of included patients, nature of primary outcome, number of deaths in men, number of deaths in women, adjusted odds ratio, factors used in multivariable analysis (where reported), number of MCS devices used in men and women and nature of MCS device. Subgroup analyses were performed for aetiology of cardiogenic shock and type of mechanical circulatory support, with sensitivity analyses including only studies at lower risk of bias.

Due to the predicted high heterogeneity of included studies, we performed meta-analysis using the DerSimonian–Laird random effects model with inverse variance weighting. Data are reported as odds ratios (ORs) with 95% confidence intervals. *P* values for the main analyses were adjusted for multiple comparison using the Holm–Bonferonni correction.

Meta-regression was conducted using the Begg–Mubazzar test and Egger’s regression analysis to assess for publication bias; in addition, funnel plots were visually inspected.

All statistical analysis was undertaken in R version 4.1.1. (R Foundation for Statistical Computing, Vienna, Austria) using package ‘metafor’ (Viechtbauer 2010).

## Results

In total, 81 studies reporting at least one sex-specific outcome were included in meta-analysis [12–16, 21–98], comprising data from eight randomised controlled studies and 73 observational cohort studies. The pooled studies contained data on 656,754 women and 1,018,036 men.

**Fig. 1:**
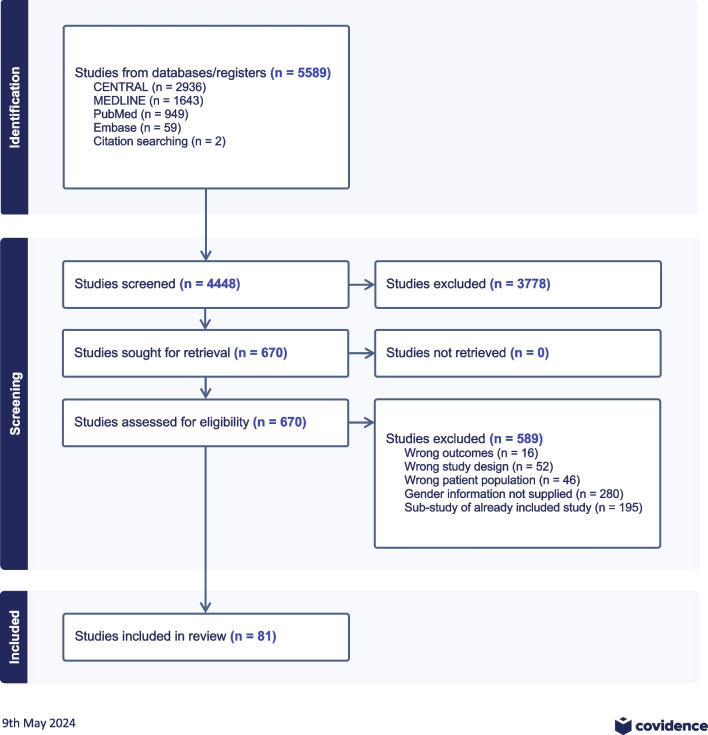
PRISMA flow diagram for epidemiological meta-analysis of sex differences in cardiogenic shock

A flow diagram for the meta-analysis is given in Fig. 1. Of 4448 abstracts screened, 670 progressed to full-text review. 589 were excluded (sex-specific outcomes not reported, n = 280; multiple publications from same study or dataset, n = 195; wrong study design n = 52; wrong patient population (e.g. extracorporeal cardiopulmonary resuscitation, post-cardiotomy) n = 46; wrong outcomes n = 16). Fifteen studies were identified from the United States National Inpatient Sample with overlapping enrolment and criteria. As in the methods above, only the largest study was included [63].

### Unadjusted mortality

**Fig. 2:**
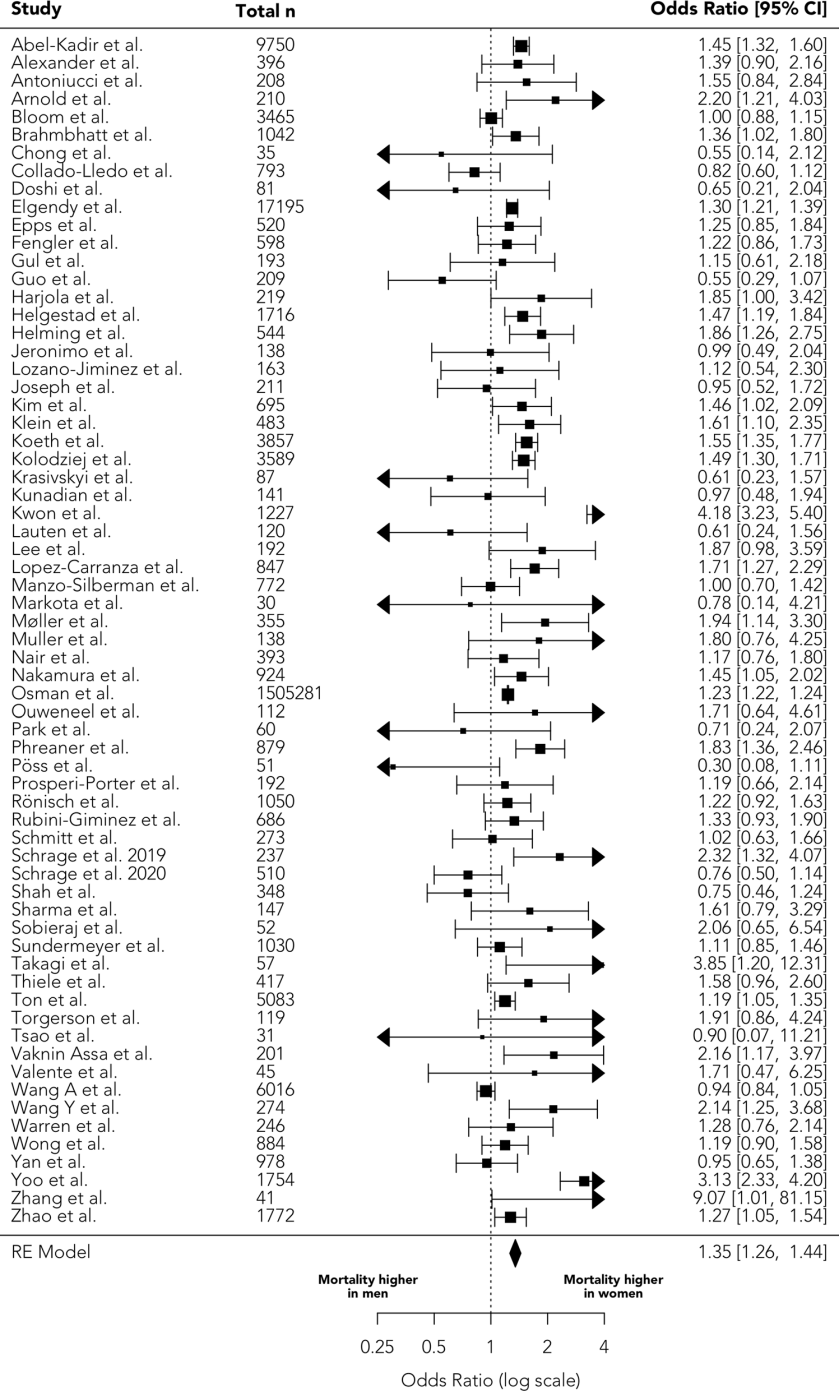
Effect of sex on unadjusted in-hospital/30-day mortality in cardiogenic shock

Unadjusted sex-specific mortality was reported in 56 studies including data for 617,801 women and 962,561 men (Fig. 2). Odds ratio for female sex and combined in-hospital and 30-day mortality was 1.35 (95% confidence interval 1.26–1.44, *p* < 0.001, τ^2^ = 0.031, I^2^ = 77.3%), Fig. 2. Pooled unadjusted mortality was 35.9% in men and 40.8% in women (*p* < 0.001).

The included data from the National Inpatient Sample (NIS) comprised 95% of the total sample size of patients; the model weight for this study accordingly was the highest at 3.9% (see Supplementary Table 1). However, sensitivity analysis excluding this study showed an almost identical result (OR = 1.34, 95% CI 1.24–1.47, *p* < 0.001).

### Adjusted mortality

**Fig. 3:**
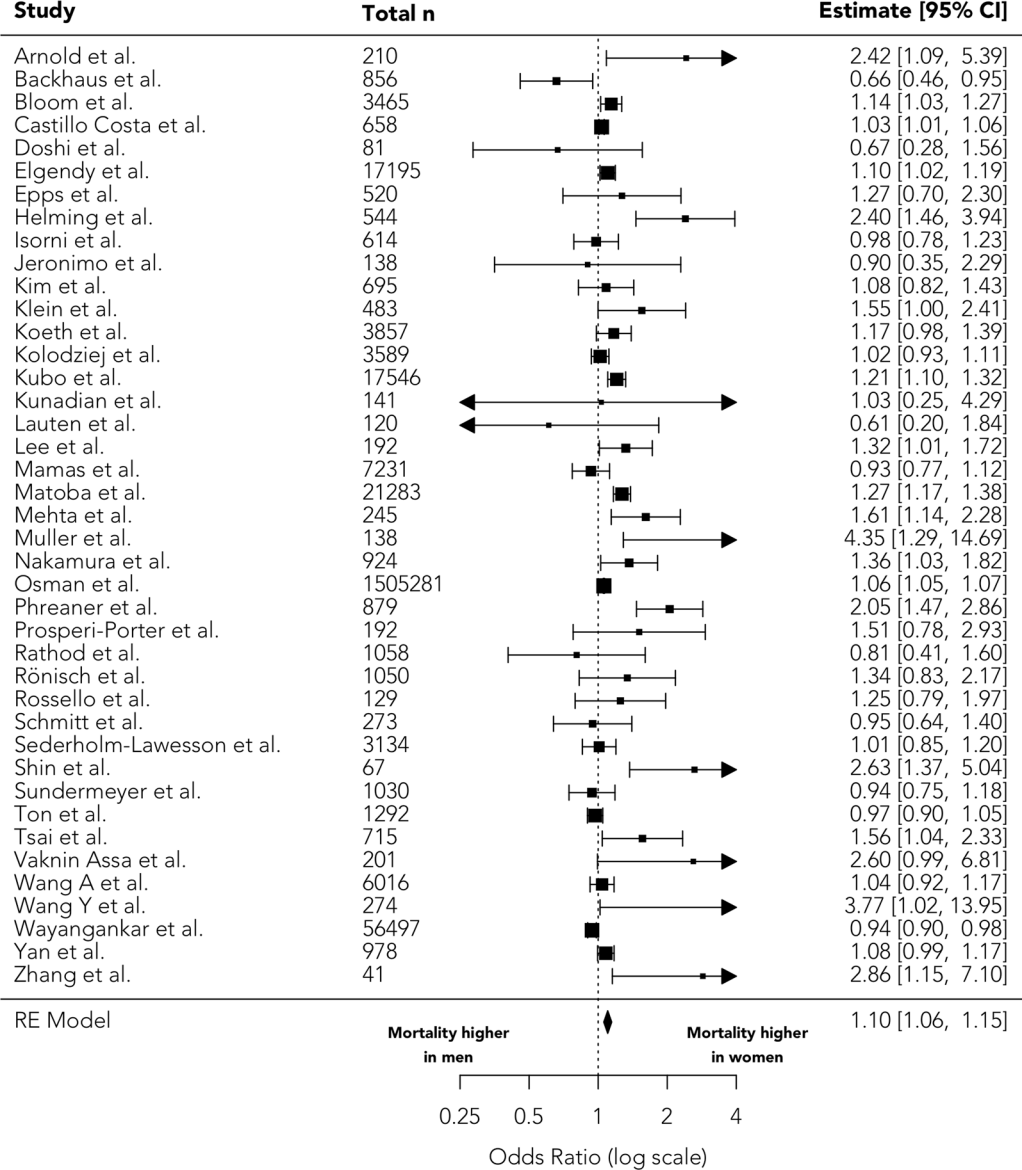
Adjusted effect of sex on in-hospital/30-day mortality in cardiogenic shock

Adjusted sex-specific mortality was available for 41 studies comprising 1,659,622 patients between 40 observational studies and a single randomised controlled trial (Fig. 3). Details of studies included in the adjusted analysis are given in Table 1. In total, 39 studies reported details of the regression model used to adjust the primary outcome. Of these, 38 studies adjusted for age, six for race/ethnicity, 26 adjusted for at least one comorbidity, and 13 for prior cardiac arrest.

**Table 1:** Characteristics of 41 studies reporting adjusted sex-specific mortality in patients with cardiogenic shock (CS)

Author	Year	Study design	Location	Population	Total, n	Factors included in model	OR
Arnold et al. [24]	2023	Observational	Switzerland	AMI-CS (STEMI)	210	Age, comorbidities, PCI, MI territory	2.42
Backhaus et al. [25]	2016	Observational	Germany	AMI-CS	856	Age, comorbidities, IABP, PCI	0.66
Bloom et al. [26]	2022	Observational	Australia	All CS	3465	Age, comorbidities, CA, PCI, STEMI, HR, SBP, MV, inotropes	1.14
Castillo Costa et al. [28]	2023	Observational	Argentina	AMI-CS (STEMI)	658	Age, CA, failed PCI	1.04
Doshi et al. [31]	2018	Observational	US	AMI-CS with pVAD	81	Age, race, comorbidities, PCI, IABP	0.67
Elgendy et al. [13]	2022	Observational	US	AMI-CS	17,195	Age, race, weight, comorbidities, STEMI	1.10
Epps et al. [32]	2023	Observational	US	All CS	520	Age, comorbidities, STEMI, lactate, RRT, inotropes	1.27
Helming et al. [39]	2014	Observational	Netherlands	AMI-CS	544	Age, comorbidities, CA	2.40
Isorni et al. [40]	2018	Observational	France	AMI-CS	614	Age, comorbidities, PCI, STEMI	0.98
Jeronimo et al. [41]	2020	Observational	Spain	All CS	138	Age, prior MI, lactate, inotropes, LVEF	0.90
Kim et al. [42]	2023	Observational	South Korea	AMI-CS with pPCI	695	Age, BMI, comorbidities, MI territory, LVEF, RRT, MV, inotropes	1.08
Klein et al. [43]	2005	Observational	US	AMI-CS	483	Age, comorbidities, MI territory, PCI, GPI	1.55
Koeth et al. [44]	2009	Observational	Germany	AMI-CS	3857	Age, comorbidities, DTBT	1.17
Kolodziej et al. [45]	2016	Observational	Poland	AMI-CS (STEMI)	3589	Age, BMI, comorbidities, CA, DTBT, SBP, HR, ECG	1.02
Kubo et al. [47]	2019	Observational	Japan	AMI-CS with pPCI	17,546	Age, comorbidities, CA, MI territory, STEMI, TRA	1.21
Kunadian et al. [48]	2013	Observational	UK	AMI-CS with pPCI	141	Age, comorbidities, CA, MI territory, TRA, DTBT, MV, GPI, IABP	1.03
Lauten et al. [50]	2012	Observational	Europe	AMI-CS with Impella	120	Age, comorbidities, CA, SBP, lactate, IABP	0.61
Lee et al. [51]	2024	Observational	New Zealand	AMI-CS (STEMI)	192	Age, comorbidities, CA, MI territory, GPI, TRA	1.32
Mamas et al. [53]	2014	Observational	UK	AMI-CS with pPCI	7231	Age, comorbidities, STEMI, LVEF, TRA, inotropes	0.93
Matoba et al. [56]	2021	Observational	Japan	AMI-CS	21,283	Age, CA, centre procedural volume	1.27
Mehta et al. [57]	2023	Observational	US	All CS on ECMO	245	Age, comorbidities, HCT, SCAI class	1.61
Muller et al. [59]	2016	Observational	France	AMI-CS on ECMO	138	Age, BMI, comorbidities, GCS, AKI, PT, lactate	4.35
Nakamura et al. [62]	2023	Observational	Japan	AMI-CS on Impella	924	Age, BMI, ECMO use, PCI	1.37
Osman et al. [63]	2021	Observational	US	All CS	1,505,281	Age, race, comorbidities, aetiology	1.06
Phreaner et al. [66]	2020	Observational	US/Canada	HF-CS	879	Age, SOFA score	2.05
Prosperi-Porter et al. [68]	2022	RCT	Canada	All CS	192	Age, SBP, inotropes, aetiology	1.51
Rathod et al. [69]	2020	Observational	UK	AMI-CS with pPCI	1058	Age, race, comorbidities, LVEF, PCI, GPI, TRA	0.81
Röhnisch et al. [70]	2018	Observational	Germany	AMI-CS	1050	Age, comorbidities, STEMI, LVEF, PCI, DTBT	1.34
Rossello et al. [71]	2016	Observational	Spain	All CS	129	Age, CS at admission, PAC use	1.25
Schmitt et al. [73]	2023	Observational	Germany	All CS	273	Age, BMI, aetiology, CA, lactate, AKI, LVEF, inotropes	0.95
Sederholm-Lawesson et al. [76]	2019	Observational	Sweden	AMI-CS (STEMI)	3134	PCI	1.01
Shin et al. [79]	2021	Observational	South Korea	AMI-CS on ECMO	67	Age, ECMO duration	2.63
Sundermeyer et al. [82]	2024	Observational	Europe	HF-CS	1030	Age, CA, SCAI class, lactate, MV	0.94
Tsai et al. [87]	2022	Observational	Taiwan	AMI-CS	715	Age, ECMO, PCI	1.56
Ton et al. [12]	2023	Observational	US	All CS	5083	Age, BMI, comorbidities, aetiology, MAP, HR, lactate	0.97
Vaknin Assa et al. [89]	2020	Observational	Israel	AMI-CS	201	NR	2.60
Wang et al. [91]	2022	Observational	International	All CS on ECMO	6016	Age, race, BMI, comorbidities, CA, RRT, aetiology	1.04
Wang Y et al. [92]	2022	Observational	China	AMI-CS (STEMI)	274	Age, glucose, PCI, LVEF, IABP	3.77
Wayangankar et al. [94]	2016	Observational	US	AMI-CS with pPCI	56,497	Age, race, BMI, comorbidities, IABP, STEMI, hospital type, LVEF	0.94
Yan et al. [15]	2021	Observational	Germany	All CS	978	Age, comorbidities, CA, CPR duration, lactate, SI, LVEF,	1.08
Zhang et al. [97]	2014	Observational	US	AMI-CS on ECMO	41	NR	2.86

AMI-CS = cardiogenic shock due to acute myocardial infarction, HF-CS = cardiogenic shock due to decompensated heart failure, STEMI = ST-elevation myocardial infarction; pVAD = percutaneous ventricular assist device. (p)PCI = (primary) percutaneous coronary intervention; ECMO = extracorporeal membrane oxygenation; IABP = intra-aortic balloon pump; CA = cardiac arrest; HR = heart rate; SBP = systolic blood pressure; MV = mechanical ventilation; RRT = renal replacement therapy; LVEF = left ventricular ejection fraction; GPI = glycoprotein IIa/IIIa inhibitor; DTBT = door-to-balloon time; ECG = electrocardiogram changes; MAP = mean arterial pressure; TRA = transradial access, BMI = body mass index; HCT = haematocrit; AKI = acute kidney injury; SCAI = Society for Cardiovascular Angiographic Intervention; PAC = pulmonary artery catheter; NR = not reported

The pooled adjusted odds ratio for female sex and combined in-hospital/30-day mortality was 1.10 (95% CI 1.06–1.15, *p* < 0.001, τ^2^ = 0.005, I^2^ = 74.22%), Fig. 3. Whilst the largest study from the NIS again comprised the majority of the sample (90.7%), it was weighted at 8.3% in the meta-analysis, and sensitivity analysis performed with the exclusion of this study demonstrated a similar result (OR = 1.13, 95% CI 1.07–1.19, *p* < 0.001, τ^2^ = 0.012, I^2^ = 74.58%).

### Mechanical circulatory support

**Fig. 4:**
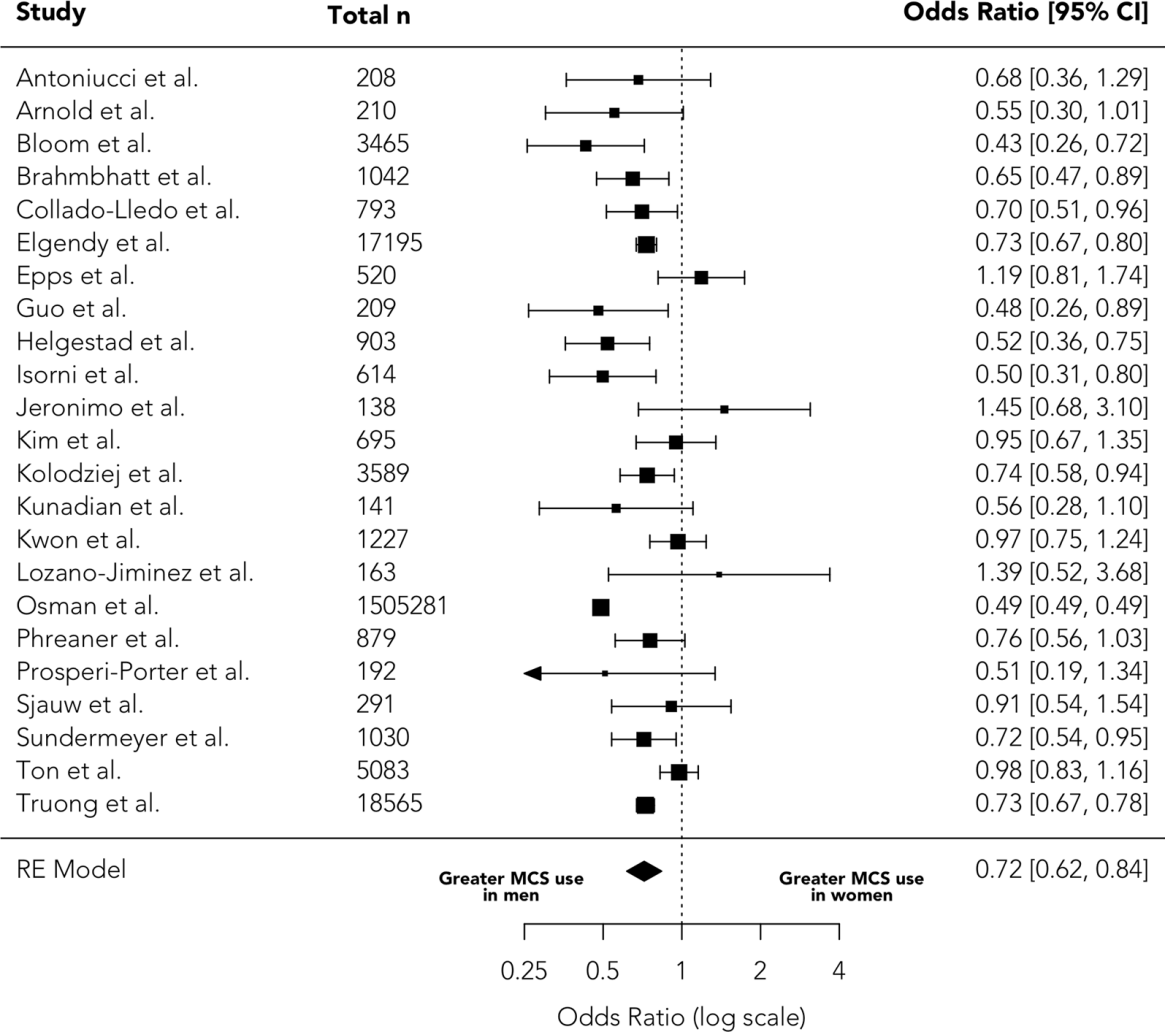
Sex-specific use of mechanical cardiac support in cardiogenic shock

Sex-specific use of MCS was reported in 23 studies with a total of 1,559,978 patients. Overall, women were less likely to receive MCS (OR = 0.72 (95% CI 0.62–0.84, *p* < 0.001, τ^2^ = 0.10, I^2^ = 93.67%), Fig. 4.

Nineteen studies reported sex-specific use of intra-aortic balloon pump (IABP), 12 reported sex-specific use of extracorporeal membrane oxygenation (ECMO) and 9 reported percutaneous ventricular assist device (pVAD). Among these, women were less likely to be treated with IABP (OR = 0.79, 95% CI 0.71–0.89, *p* < 0.001, τ^2^ = 0.02, I^2^ = 65.28%) and ECMO (OR = 0.84, 95% 0.71–0.99, *p* = 0.045, τ^2^ = 0.04, I^2^ = 62.11% There was no significant difference seen in the use of percutaneous VAD (OR = 0.82, 95% CI 0.51–1.33, *p* = 0.42, τ^2^ = 0.39, I^2^ = 96.06%).

### Heterogeneity, bias, and sensitivity analysis

Visual inspection of funnel plots (Supplementary Fig. 1a) suggested low likelihood of publication bias for the unadjusted mortality analyses, subsequently confirmed by Begg’s (τ =  − 0.10, *p* = 0.23), and Egger’s tests (*p* = 0.62). For the adjusted mortality analysis, visual inspection of the funnel plot (Supplementary Fig. 1b) suggested moderate likelihood of publication bias, with the missing effect estimates of significantly increased mortality in women. This was not significant by Begg’s test (τ = 0.15, *p* = 0.16) but was by meta-regression (Egger’s test *p* < 0.001). The MCS analysis (Supplementary Fig. 1c) showed moderate likelihood of publication bias by Begg’s (τ =  − 0.38, *p* = 0.012) but not Egger’s tests (*p* = 0.88).

As discussed above, significant heterogeneity of studies was observed. Sensitivity analysis was performed including only studies with low risk of bias (Newcastle–Ottawa Score ≥ 7). In this sensitivity analysis (Supplementary Figs. 2–4), unadjusted OR for female sex and mortality was 1.31 (95% CI 1.22–1.41, *p* < 0.001, τ^2^ = 0.03, I^2^ = 78.9%), and adjusted OR for female sex and mortality was 1.09 (95% CI 1.05–1.13, *p* < 0.001, τ^2^ = 0.004, I^2^ = 72.5%). The OR for female sex and receipt of MCS in sensitivity analysis was 0.74 (95% CI 0.62–0.88, *p* = 0.001, τ^2^ = 0.11, I^2^ = 92.9%).

### Subgroup analyses

Two a priori subgroup analyses were specified: AMI-cardiogenic shock and heart failure cardiogenic shock (HF-cardiogenic shock). Data for 634,036 patients (254,741 females and 379,295 males) with AMI-cardiogenic shock from 45 studies were available. In females with AMI-cardiogenic shock, the unadjusted OR was 1.45 (95% CI 1.34–1.56, *p* < 0.001, τ^2^ = 0.018, I^2^ = 64.8%). Adjusted OR for female sex and in-hospital/30-day mortality in AMI-cardiogenic shock was 1.13 (95% CI 1.06–1.22, *p* < 0.001, τ^2^ = 0.013, I^2^ = 77.8%), (Supplementary Figs. 5 and 6).

Data were available for 928,263 patients with HF-cardiogenic shock (370,215 women and 558,048 men from 8 studies. In females with HF-cardiogenic shock, the unadjusted OR was 1.36 (95% CI 1.05–1.77, *p* = 0.021, τ^2^ = 0.087, I^2^ = 85.5%), Supplementary Fig. 7. Only three studies reported an adjusted OR for HF-cardiogenic shock patients, so meta-analysis was not performed.

In a post hoc analysis including only randomised controlled trials, consisting of 721 women and 2,040 men, the unadjusted OR for female sex and mortality was 1.39 (95% CI 1.17–1.66, *p* < 0.001, τ^2^ = 0.001, I^2^ = 1.55%), Supplementary Fig. 8. Mortality was higher in women in all but one study (Table 2).

**Table 2:** Randomised controlled trials in cardiogenic shock reporting sex-specific mortality and risk ratios for intervention

Name	Year	Comparison	Enrolment		Mortality		RR for mortality with intervention		
			Men (%)	Women (%)	Men (%)	Women (%)	All	Men	Women
TRIUMPH [22]	2007	Tilarginine acetate vs placebo	72	28	43	51	1.14 (0.92–1.41)	1.12 (0.85–1.46)	1.17 (0.82–1.69)
IABP-SHOCK-II [33]	2012	IABP vs standard care	69	31	39	44	0.96 (0.79–1.17)	0.92 (0.72–1.18)	1.03 (0.74–1.43)
CULPRIT-SHOCK [72]	2020	Culprit vs complete revascularisation	76	24	49	56	0.84 (0.72–0.98)	0.76 (0.64–0.91)	1.02 (0.77–1.35)
OptimaCC [83]	2020	Noradrenaline vs adrenaline	67	33	26	57	8.24*(1.61–42.2)	NR	NR
DOREMI [68]	2021	Dobutamine vs milrinone	64	36	50	54	0.90 (0.69–1.19)	0.94 (0.66–1.34)	0.85 (0.55–1.31)
ECLS-SHOCK [84]	2023	ECMO vs standard care	81	19	46	57	0.98 (0.80–1.19)	0.98 (0.81–1.20)	0.94 (0.56–1.58)
EVOLVE-ECMO [65]	2023	LV unloading + ECMO vs ECMO	65	35	51	42	0.91** (0.67–1.24)	1.13 (0.78–1.63)	0.61 (0.34–1.08)
DANGER-SHOCK [58]	2024	pVAD vs. standard care	79	21	49	65	0.74 (0.55–0.99)	0.66 (0.47–0.93)	1.01 (0.58–1.72)

RR = relative risk, NR = not reported, IABP = intra-aortic balloon pump, LV = left ventricle, ECMO = extracorporeal membrane oxygenation, pVAD = percutaneous ventricular assist device

* = RR is for refractory shock, trial stopped early due to high incidence in adrenaline group. ** = RR is for successful ECMO weaning

## Discussion

This is the first systematic review examining sex differences in outcome in patients with cardiogenic shock. Strengths of this study include geographically representative data, a large sample size and well-defined outcome measures (in-hospital mortality and use of MCS). The observed effect size remained present after exclusion of studies with a high risk of bias, and we undertook a rigorous process to limit inclusion of multiple publications from the same patient dataset. Our key findings are that, after adjustment for baseline characteristics, female patients with cardiogenic shock were 10% more likely to die than male patients, and 30% less likely to receive MCS. The observed difference in mortality was mirrored in sub-group analyses of AMI-cardiogenic shock and HF-cardiogenic shock.

There are multiple potential explanations for our findings, which include: (1) persistent confounding from variables not adjusted for in studies reporting adjusted analysis; (2) differences in cardiogenic shock aetiology and pathophysiology between men and women; (3) sex-related healthcare behaviours and/or systemic bias from clinicians leading to women presenting at a more advanced stage of cardiogenic shock; (4) differential therapeutic effects causing women to derive less benefit from treatments than men; and (5) implicit bias from treating healthcare professionals leading to less aggressive treatment of cardiogenic shock in women.

Included studies reported that women who develop cardiogenic shock are older [13, 15, 21, 26, 48, 63] and have more comorbidities [13, 26, 63, 72]. Men with AMI-cardiogenic shock are more likely to have obstructive coronary disease, whereas women are more likely to have non-obstructive coronary arteries with other comorbidities, which may predispose them to poorer outcomes [99]. Despite men representing most patients with AMI-cardiogenic shock, women have a higher risk of developing cardiogenic shock post-AMI compared to men. Women with obstructed coronary arteries may have more diffuse disease less amenable to angiographic intervention [100]. Given that successful revascularisation of the culprit vessel(s) is strongly associated with survival in AMI-cardiogenic shock [101], this may contribute to its increased incidence and mortality in women. Sex differences in heart failure aetiology and in cardiac function have also been described in patients with chronic heart failure [102].

It is likely that women have different aetiologies of cardiogenic shock than men. Mechanical complications of AMI such as ventricular septal defect formation and papillary muscle rupture are more common in women [103]. Several non-AMI causes of de novo heart failure are more common in women, including Takutsubo syndrome, peripartum cardiomyopathy, myocarditis and valvular aetiologies [4, 15, 33, 41, 104]. Although outcomes in different de novo aetiologies vary, with better outcomes in patients with CS secondary to Takotsubo syndrome [105], recent registry data report higher Sequential Organ Failure Assessment (SOFA) scores and more common presentations of SCAI E stage of CS in de novo CS compared to acute-on-chronic HF-CS, with higher associated in-hospital mortality [106]. This may be attributed to the absence of validated treatments assessed in large randomised trials to manage such patients.

Women with cardiogenic shock may have greater shock severity at presentation. Female patients have lower systolic and diastolic blood pressure on presentation, and across all aetiologies are more likely to present in the most severe (*Extremis)* Society for Cardiovascular Angiography and Interventions (SCAI) stage of cardiogenic shock [14, 15, 33, 41]. This finding itself is likely multifactorial: whilst contrary to some traditional teaching, women are equally likely as men to experience ‘classical’ symptoms of STEMI with chest pain, they may be less likely to perceive these as those of a heart attack [107]. Women are less likely to be correctly diagnosed with STEMI pre-hospital and triaged to an appropriate hospital [108], and have longer symptom-to-balloon time and door-to-balloon time than men [109]. Delay in recognition of cardiogenic shock as the cause of deterioration in women is likely to correlate with worse physiological derangement at presentation.

Less aggressive treatment of women with cardiogenic shock may lead to higher mortality. Women with AMI-cardiogenic shock have been shown to be less likely to receive revascularisation [11, 109]. The data herein demonstrate that women with all-cause cardiogenic shock are less likely to receive MCS. Clinicians may be less likely to pursue MCS in women with cardiogenic shock due to their baseline differences including older age, comorbid disease and increased frailty. In some jurisdictions, women with cardiogenic shock are more often from more deprived socioeconomic groups, which may consciously or unconsciously affect decision-making [110].

The evidence base for treatment of cardiogenic shock derives almost entirely from men: the proportion of women enrolled in the eight included RCTs (Table 2) ranges from 19 to 35%. In the three trials to date which have reported a survival benefit from an intervention in AMI-related cardiogenic shock, the benefit was seen only in the male patients. In DANGER-SHOCK, the relative risk for mortality with a pVAD was 1.01 (0.58–1.79) in women compared to 0.67 (0.47–0.93) for men [58]; in CULPRIT-SHOCK the relative risk for mortality with culprit vs. complete revascularisation in women was 1.02 (0.77–1.35) compared to 0.76 (0.64–0.91) in men [72]. The SHOCK trial (revascularisation vs. medical therapy) was not included in our analysis as sex-specific mortality data was unavailable, but also reported a statistically significant benefit for revascularisation in men but no significant benefit in women with a relative risk > 1 [111].

It is possible that these discrepancies are due to the low enrolment of women in RCTs and hence inadequate statistical power to detect benefit of these interventions in women. However, there could also be factors which cause women to derive less benefit from revascularisation and/or MCS than men. Revascularisation may be more challenging in women for the reasons listed above. Complications of MCS, specifically bleeding and limb ischaemia, occur more frequently in women [112, 113], who are more likely to have smaller vessel size, smaller body surface area, and altered haemocompatibility. These factors may contribute to conscious or unconscious bias against the provision of advanced therapies to women.

There are further potential systemic biases against female patients in acute cardiovascular care. Several guidelines for escalation of therapy in cardiogenic shock use haemodynamic and biochemical thresholds extracted from analysis of databases with predominantly male patients. As demonstrated, women are underrepresented in cardiovascular disease trials [114], the results of which eventually comprise the basis of clinical guidelines including the SCAI classification of CS. The disparity in the outcome between male and female sex has been noted in the SCAI consensus on sex specific considerations in myocardial revascularisation with recommendations to create sex-based algorithms [115]. It may be that, given the results of our study, a similar approach is required to address disparities in evidence and in receipt of treatment, promote awareness of cardiovascular disease in women and to close the “sex gap” in cardiogenic shock [116].

### Limitations

Several factors limit the conclusions we can draw from our meta-analysis. Our inclusion of studies from over a twenty-year period led to a large cohort, however this may limit applicability to current practice particularly as cardiogenic shock definitions and therapeutic strategies have evolved over this period.

Although included studies originated from six continents, the majority of our data is derived from countries with access to advanced resources with established preventive public health policies. Most included studies were conducted in tertiary cardiac centres. There was limited evidence from non-specialised units which limits applicability of our findings to this setting. Most included studies assessed patients with AMI- cardiogenic shock, which is not reflective of the contemporary increasing prevalence of cardiogenic shock due to other causes. There were insufficient data to perform a meta-analysis on HF-cardiogenic shock adjusted mortality. We were unable to access individual patient data for the included studies, and thus were unable to stratify data by age, race, or comorbidities which may have limited the conclusions we can draw regarding causes of the observed differences in women and men.

Our meta-analysis on adjusted mortality is limited by moderate likelihood of publication bias, however, if reported, the missing studies would in fact strengthen the observed effect size. Given the lack of an international consensus definition of cardiogenic shock, inclusion criteria and definition of cardiogenic shock definitions may have differed among included studies. The reliability of our mortality analysis is limited by inconsistent definitions of mortality used by included studies. Our mortality endpoint comprises a combination of in-hospital and 30-day mortality. Furthermore, our meta-analysis does not provide information about long-term morbidity and mortality or other patient-centred outcomes.

## Conclusions

This systematic review and meta-analysis found increased in-hospital mortality and lower temporary MCS use in cardiogenic shock amongst female patients when compared to male patients. While this may be due to women presenting later in the disease course and a higher comorbidity burden, we cannot exclude the effect of implicit clinician bias. Further research is required to address the causes of this disparity and on how outcomes can be improved to ensure equitable management and access to therapies for women.

### Supplementary Information

Supplementary Material 1

Supplementary Material 2

## Data Availability

The datasets generated are publicly available in our Supplementary Data File.
