# Supplementary material for: Sex differences in treatments and outcomes of patients with cardiogenic shock: a systematic review and epidemiological meta-analysis
Source: Crit Care. 2024 Jun 6;28:192. doi: 10.1186/s13054-024-04973-5 (PMC11157877; doi:10.1186/s13054-024-04973-5)

Supplementary Table 1
Model weights for a meta-analysis of effect of sex on unadjusted 30-day/inpatient mortality in cardiogenic shock.

Abel-Kadir et al.	Alexander et al.	Antoniucci et al.	Arnold et al.
3.58464949	1.47624159	0.94807701	0.95790575
Bloom et al.	Brahmbhatt et al.	Chong et al.	Collado-Lledo et al.
3.33984301	2.30989113	0.23552635	2.12502762
Doshi et al.	Elgendy et al.	Epps et al.	Fengler et al.
0.32260228	3.72151084	1.71149167	1.90603537
Gul et al.	Guo et al.	Harjola et al.	Helgestad et al.
0.87600151	0.83438605	0.92880714	2.76069973
Helming et al.	Jeronimo et al.	Lozano-Jiminez et al.	Joseph et al.
1.69568419	0.72987160	0.72384352	0.96749092
Kim et al.	Klein et al.	Koeth et al.	Kolodziej et al.
1.85643131	1.75054989	3.35683633	3.33187074
Krasivskyi et al.	Kunadian et al.	Kwon et al.	Lauten et al.
0.45021339	0.76104648	2.49508142	0.46254474
Lee et al.	Lopez-Carranza et al.	Manzo-Silberman et al.	Markota et al.
0.85349340	2.24851013	1.88385904	0.15547927
Møller et al.	Muller et al.	Nair et al.	Nakamura et al.
1.14948259	0.53947558	1.50355751	2.02367201
Osman et al.	Ouweneel et al.	Park et al.	Phreaner et al.
3.86132426	0.42125283	0.36664107	2.21570882
Pöss et al.	Prosperi-Porter et al.	Rönisch et al.	Rubini-Giminez et al.
0.25425710	0.99041774	2.29230708	1.88435169
Schmitt et al.	Schrage et al. 2019	Schrage et al. 2020	Shah et al.
1.29305143	1.06038712	1.59004322	1.26272217
Sharma et al.	Sobieraj et al.	Sundermeyer et al.	Takagi et al.
0.73160582	0.31705264	2.38744063	0.31439326
Thiele et al.	Ton et al.	Torgerson et al.	Tsao et al.
1.25955884	3.41537424	0.60968438	0.07134762
Vaknin Assa et al.	Valente et al.	Wang A et al.	Wang Y et al.
0.94192529	0.25592161	3.51136128	1.12775841
Warren et al.	Wong et al.	Yan et al.	Yoo et al.
1.20009513	2.31870982	1.77845969	2.24551395
Zhang et al.	Zhao et al.		
0.09389713	2.94974510		

Supplementary Table 2

Arnold	Backhaus	Bloom	Castillo Costa	Doshi
0.23810574	1.04018745	5.24258640	8.05706196	0.21231918
Elgendy	Epps	Helming	Isorni	Jeronimo
6.34755638	0.42503785	0.59299607	2.28403105	0.17618177
Kim	Klein	Koeth	Kolodziej	Kubo
1.63895011	0.74091368	3.18530131	5.89867785	5.83487642
Kunadian	Lauten	Lee	Mamas	Matoba
0.07695976	0.12708651	1.75350301	2.91599265	5.98949831
Mehta	Muller	Nakamura	Osman	Phreaner
1.13188656	0.10480757	1.56745817	8.33275676	1.21374310
Prosperi-Por	Rathod	Rönisch	Rossello	Schmitt
0.34266086	0.31941563	0.62421152	0.69820970	0.90665501
Sederholm-La	Shin	Sundermeyer	Ton	Tsai
3.33634247	0.35507608	2.18094429	6.34755638	0.86649989
Vaknin Assa	Wang A	Wang Y	Wayangankar	Yan
0.16636854	4.72540107	0.09089945	7.60191373	6.12312903
Zhang				
0.18624072				

Supplementary Figure 1A

Funnel plot of publication bias for the unadjusted mortality analyses

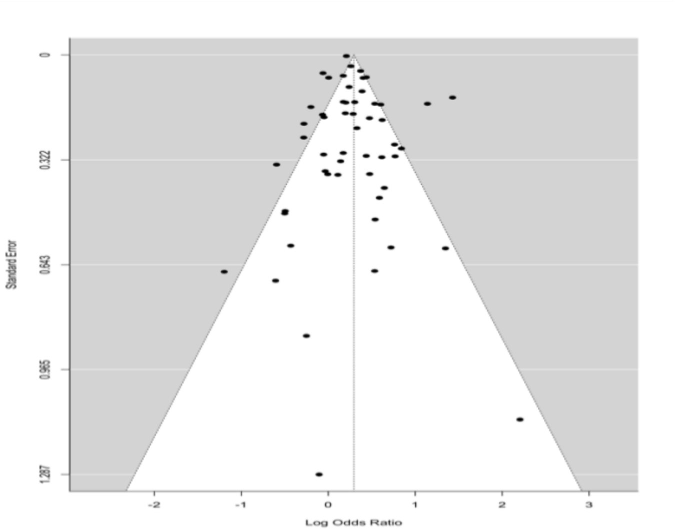

Supplementary Figure 1B

Funnel plot of publication bias for the adjusted mortality analyses

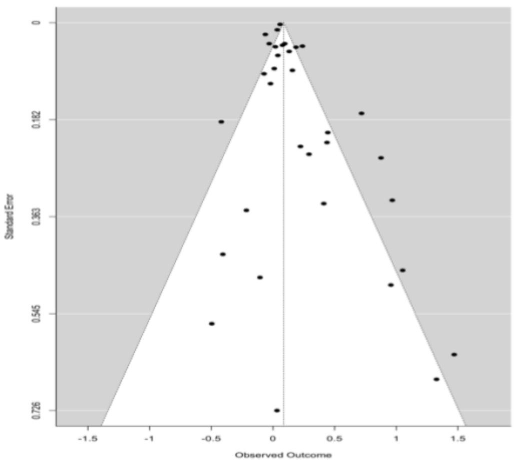

Supplementary Figure 1C

Funnel plot of publication bias for the MCS analysis

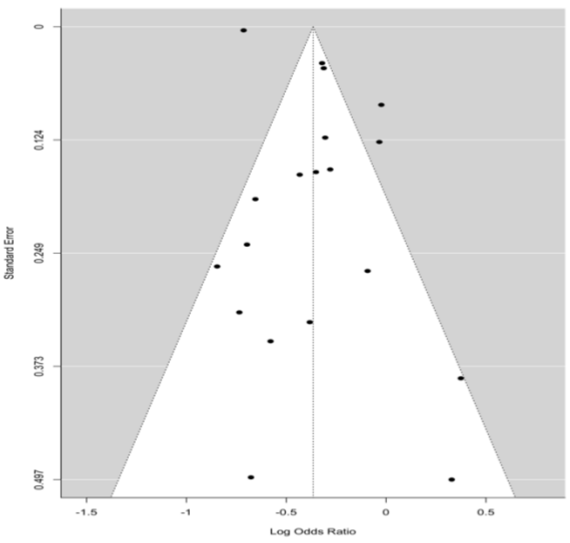

Supplementary Figure 2

Unadjusted OR for female sex and mortality

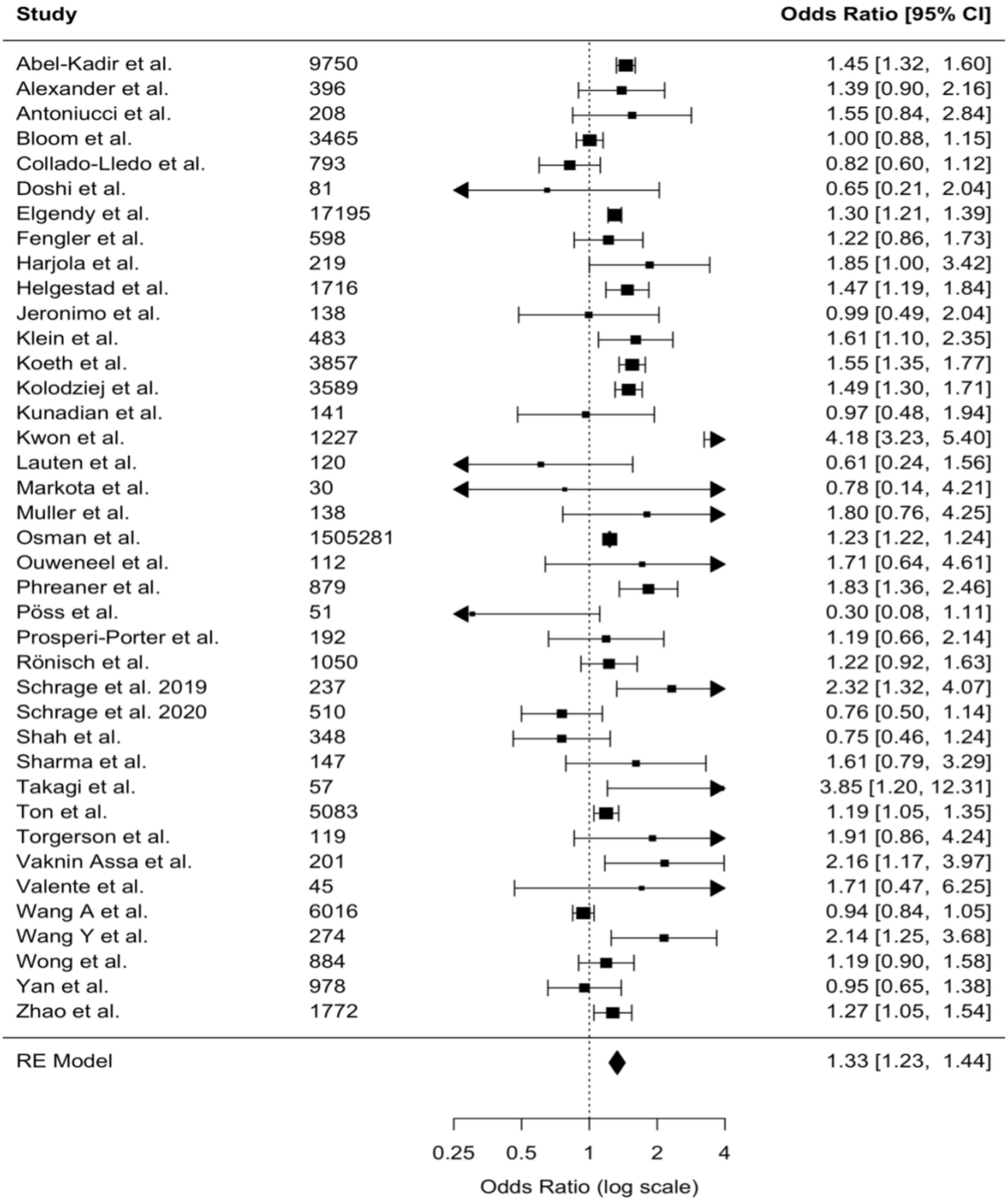

Supplementary Figure 3

Adjusted OR for female sex and mortality

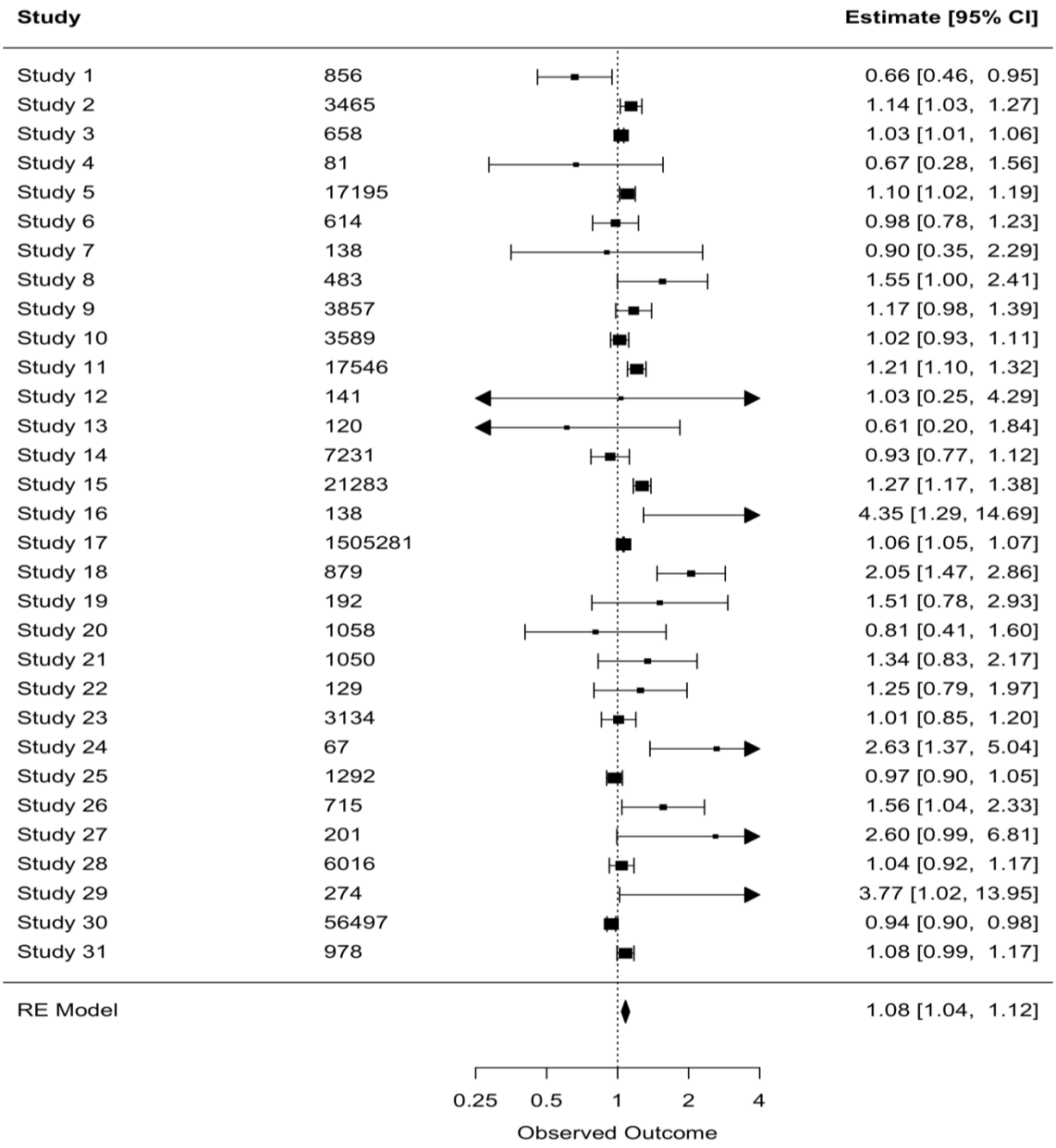

Supplementary Figure 4

Unadjusted OR for female sex and receipt of MCS

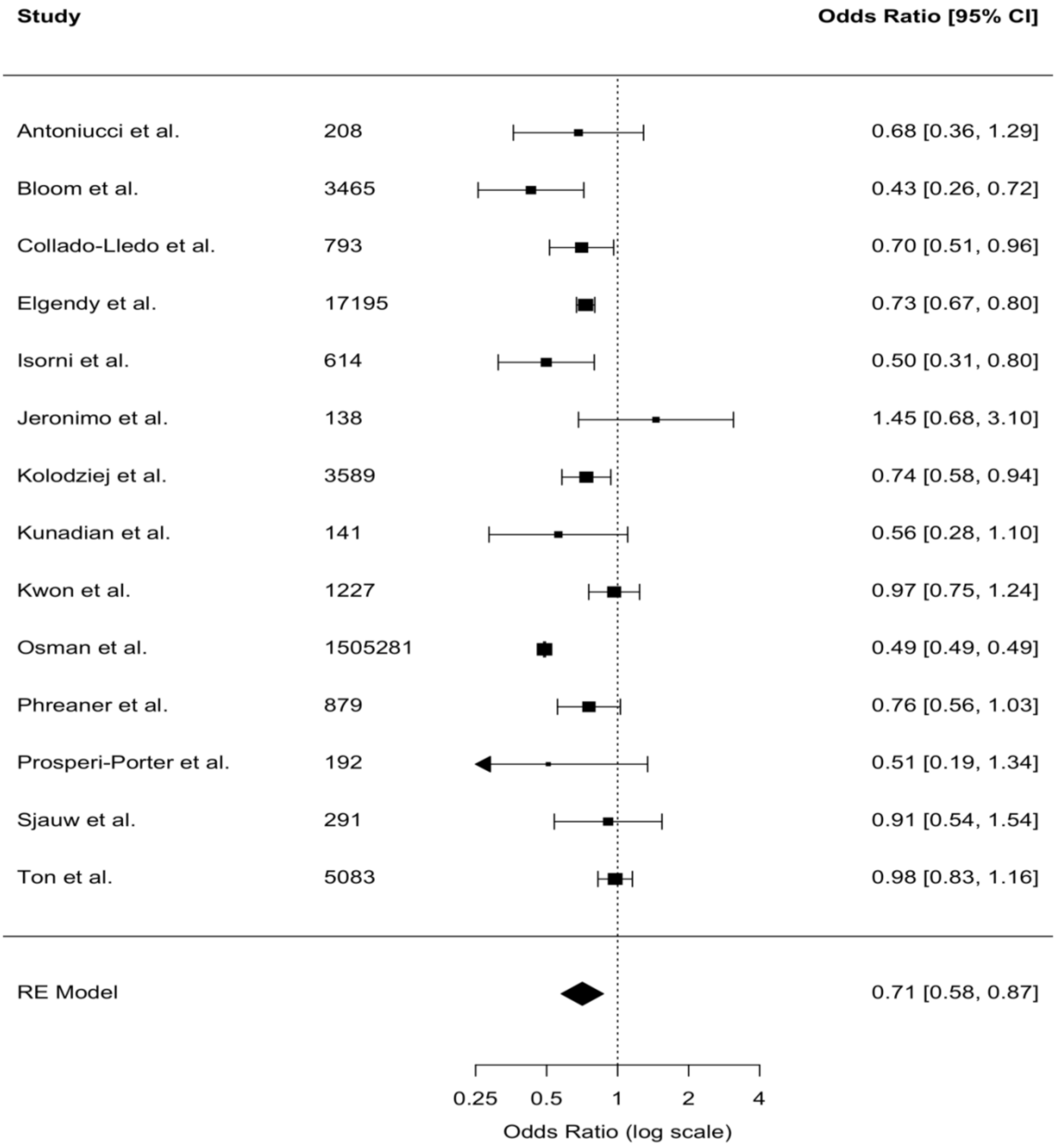

Supplementary Figure 5

Unadjusted OR for female sex and in-hospital/30-day mortality in AMI-cardiogenic shock

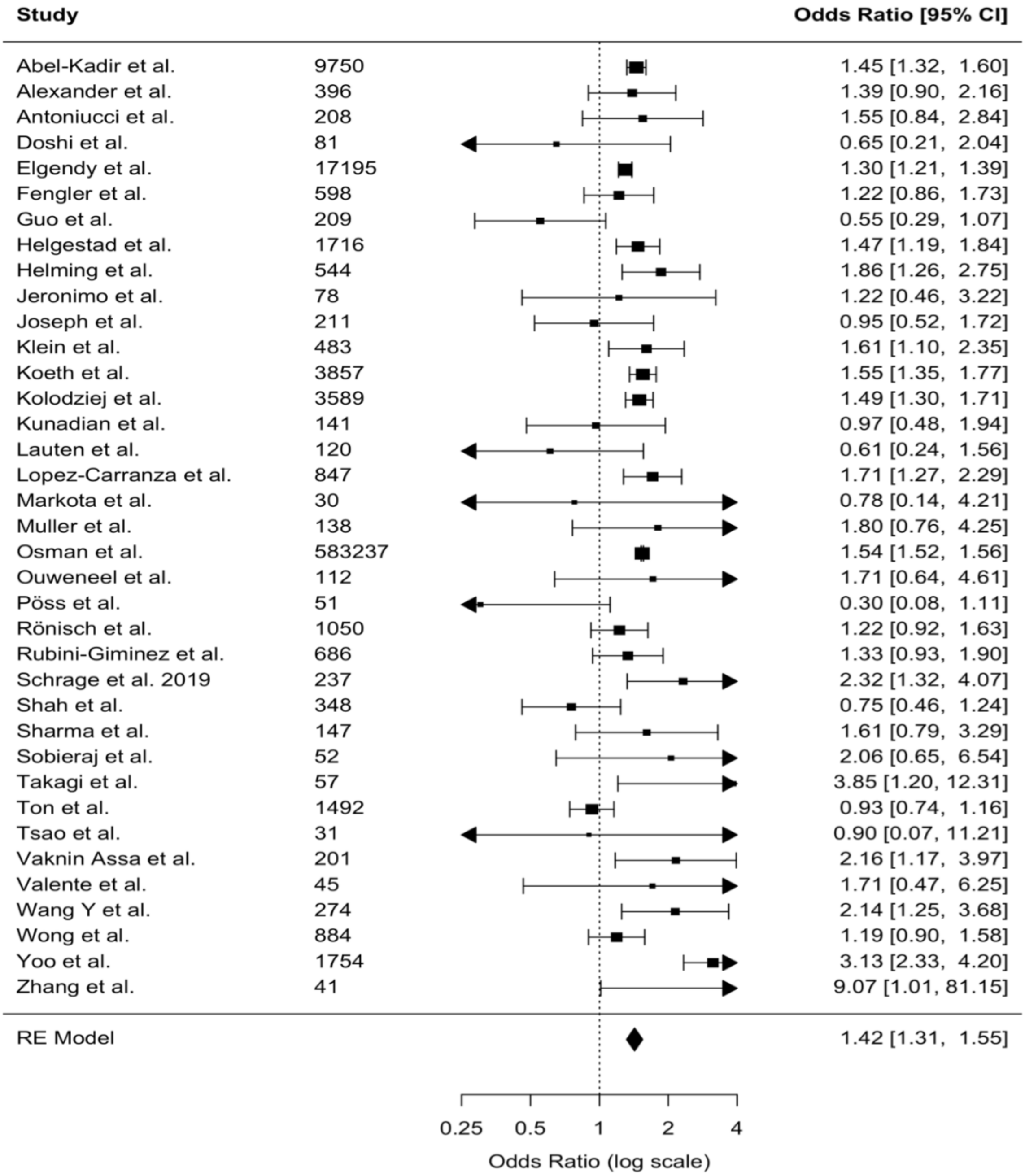

Supplementary Figure 6

Adjusted OR for female sex and in-hospital/30-day mortality in AMI-cardiogenic shock

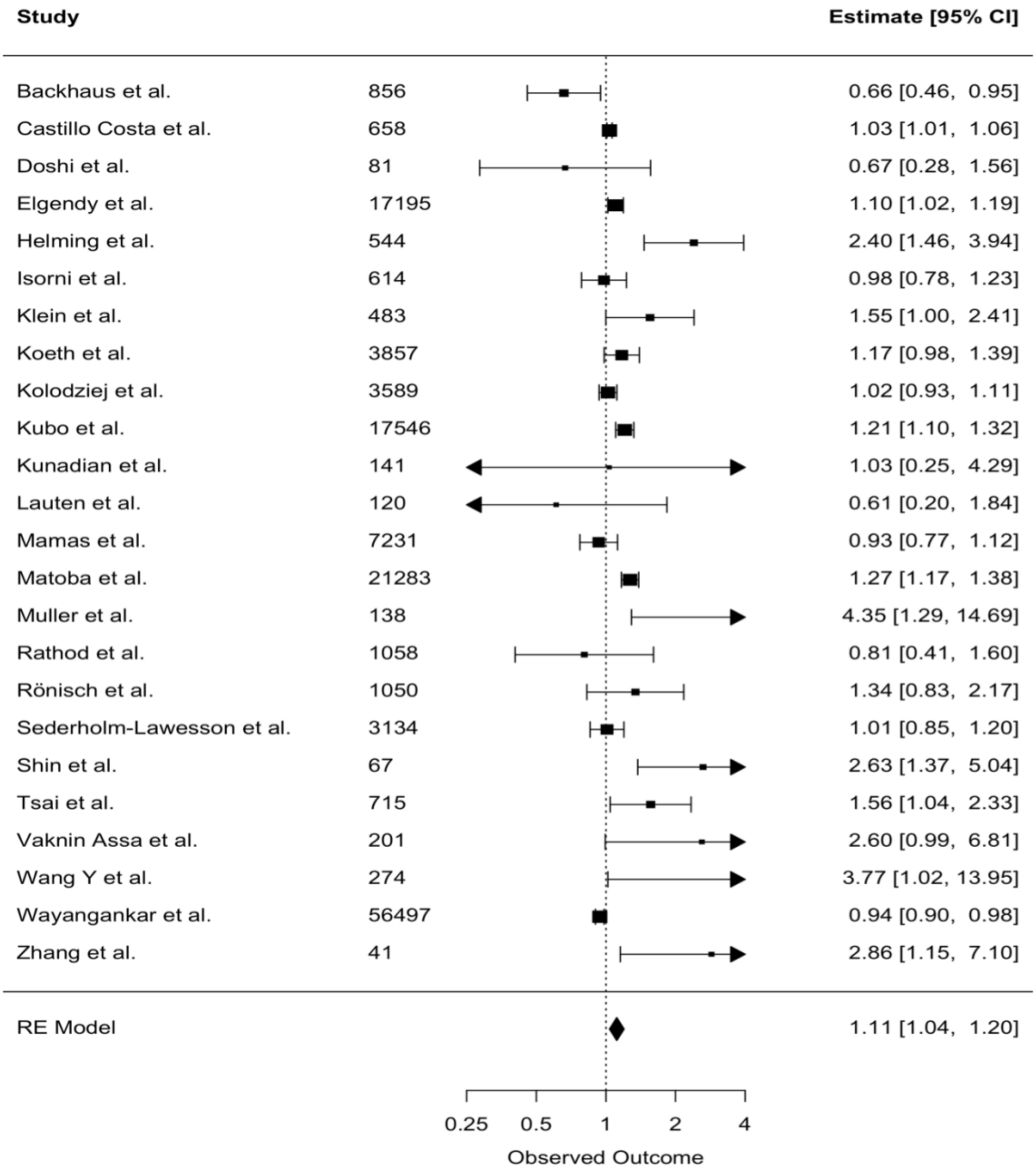

Supplementary Figure 7

Unadjusted OR for female sex and in-hospital/30-day mortality in HF-cardiogenic shock

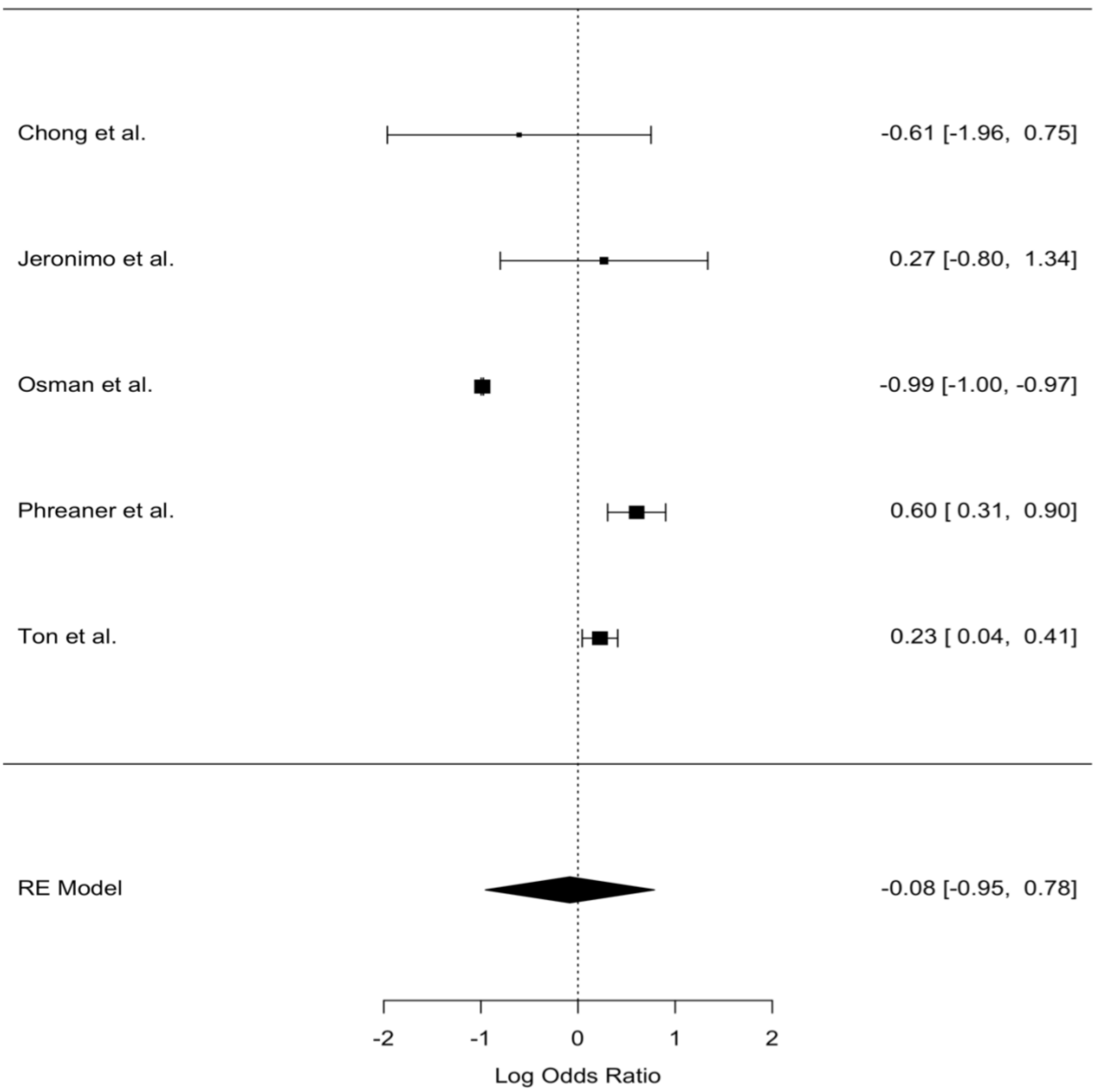

Supplementary Figure 8

Post hoc analysis of RCTs

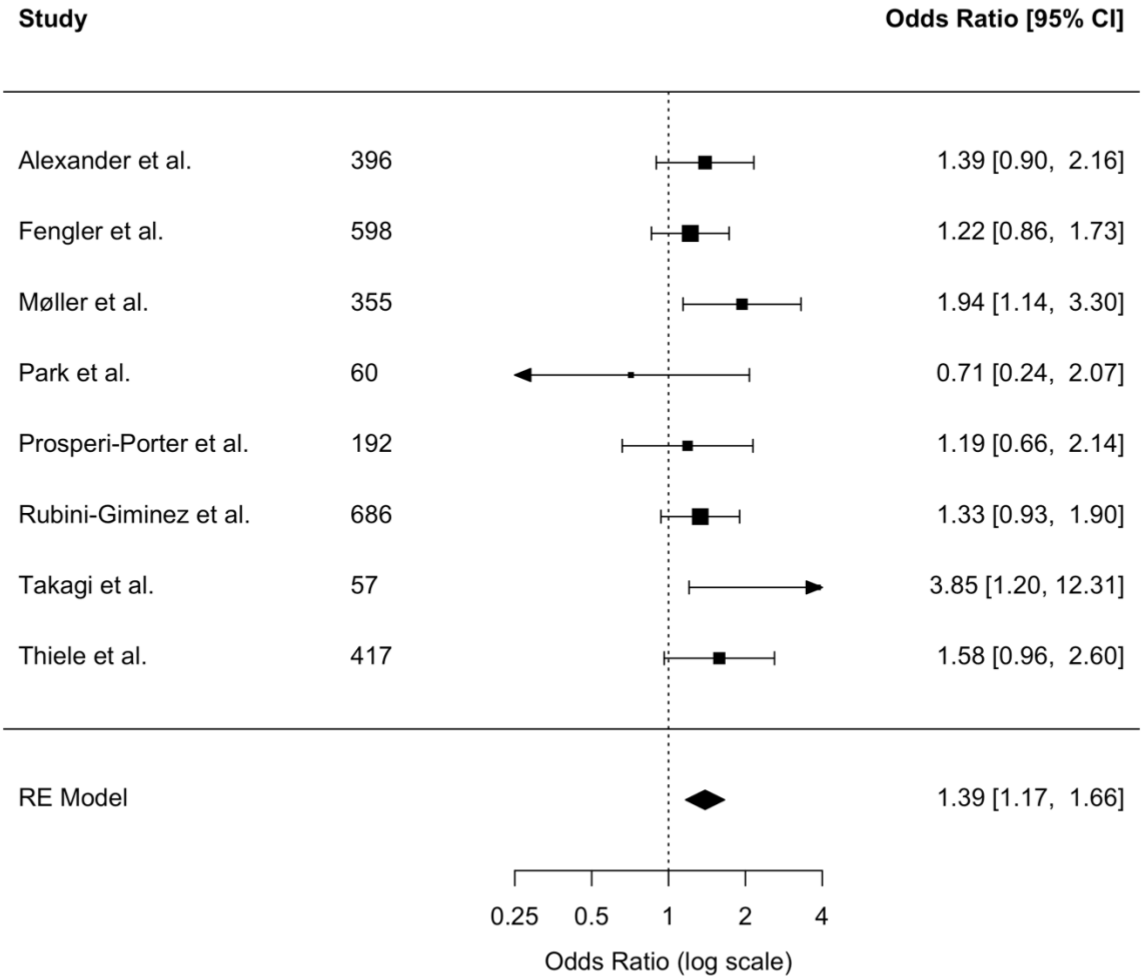

Supplement: Supplementary file 1 — Supplementary Material 1 [file 13054_2024_4973_MOESM1_ESM.pdf]
