# Supplementary material for: Sex differences in treatments and outcomes of patients with cardiogenic shock: a systematic review and epidemiological meta-analysis
Source: Crit Care. 2024 Jun 6;28:192. doi: 10.1186/s13054-024-04973-5 (PMC11157877; doi:10.1186/s13054-024-04973-5)

Supplemental Table 1.

Weights for unadjusted mortality model with and without the study by Osman et al.

1a. with


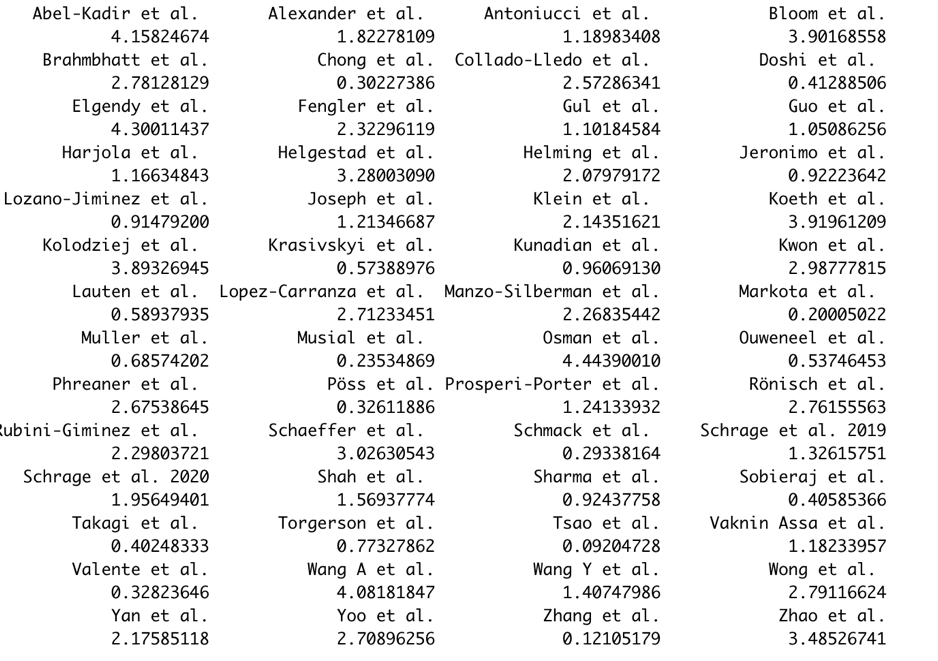


1b. without


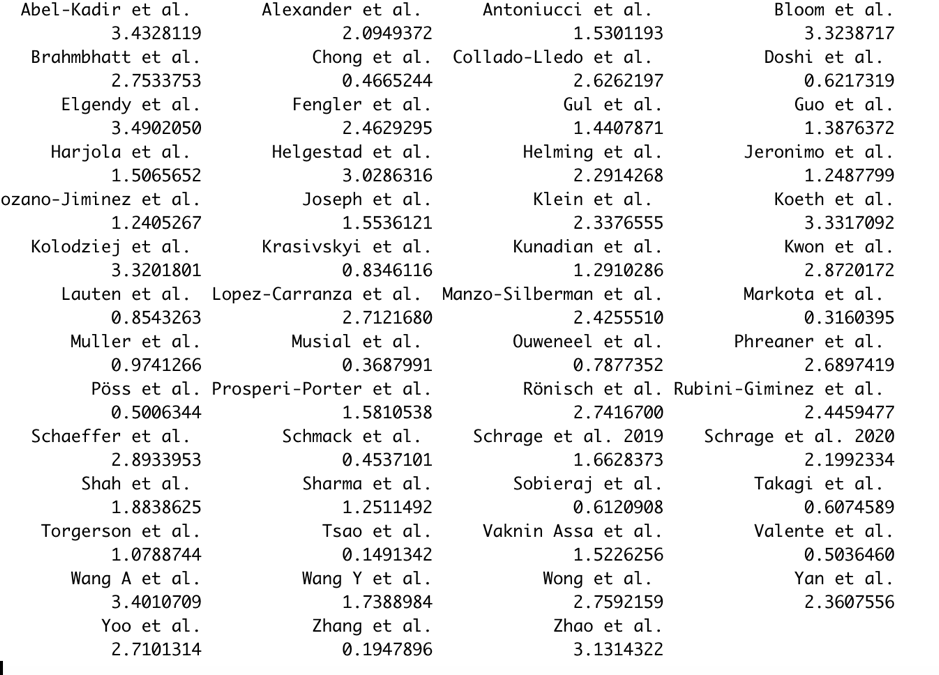

Supplement: Supplementary file 2 — Supplementary Material 2 [file 13054_2024_4973_MOESM2_ESM.docx]
